# Physical-chemical measurement method development for self-assembled, core-shell nanoparticles

**DOI:** 10.1038/s41598-018-38194-y

**Published:** 2019-02-07

**Authors:** Natalia Farkas, Puthupparampil V. Scaria, Martin C. Woodle, John A. Dagata

**Affiliations:** 1 Theiss Research, 7411 Eads Ave, La Jolla, CA 92037 USA; 2 National Institute of Standards and Technology, Gaithersburg, MD 20899 USA; 3 Aparna Biosciences, Rockville, MD 20852 USA; 4 Present Address: Laboratory of Malaria Immunology and Vaccinology, National Institute of Allergy and Infectious Diseases, National Institutes of Health, Rockville, MD 20852 USA

## Abstract

Improvements in dimensional metrology and innovations in physical-chemical characterization of functionalized nanoparticles are critically important for the realization of enhanced performance and benefits of nanomaterials. Toward this goal, we propose a multi-technique measurement approach, in which correlated atomic force microscopy, dynamic light scattering, high performance liquid chromatography and mass spectroscopy measurements are used to assess molecular and structural properties of self-assembled polyplex nanoparticles with a core-shell structure. In this approach, measurement methods are first validated with a model system consisting of gold nanoparticles functionalized with synthetic polycationic branched polyethylenimine macromolecules. Shell thickness is measured by atomic force microscopy and dynamic light scattering, and the polyelectrolyte uptake determined by chromatographic separation and mass spectrometric analysis. Statistical correlation between size, structure and stability provide a basis for extending the methods to more complex self-assembly of nucleic acids and macromolecules via a condensation reaction. From these size and analytical chemical measurements, we obtain a comprehensive spatial description of these assemblies, obtain a detailed interpretation of the core-shell evolution, and identify regions of the parameter space where stable, discrete particle formation occurs.

## Introduction

Functional nanoparticles are often realized through self-assembly, where nanometer-scale structures are formed by spatial partitioning of various components^1–3^. In core-shell structures, a payload is surrounded by a hydrophilic shell with attached molecules for target specificity^4^. Physical-chemical properties of individual nanoparticles—and their shell in particular—have key importance in achieving stability and a functional molecular structure^5^. By studying the relationship between formation and physical-chemical properties of nanoparticles, functionality of the formulation can be optimized for specific applications. Reports on *in-vitro*/*in-vivo* performance of functional nanoparticles, however, often lack systematic physical-chemical characterization and may miss important parameters that drive the self-assembly process. This practice may lead to incomplete and conflicting results on the relation between physical-chemical properties and functional efficiency. To address these issues, we present a multi-technique measurement approach to obtain quantitative dimensional, structural and compositional information of self-assembled, core-shell nanoparticles that are shown to be functionally active *in vivo*. Dynamic light scattering (DLS) is combined with atomic force microscopy (AFM), high performance liquid chromatography (HPLC) and mass spectroscopy (MS) for a systematic investigation of the formulation space at both the molecular and distributional levels.

Currently, particle size analysis of nanoparticles is dominated by—and much too frequently limited to—DLS measurements^6–9^. The complexity and polydispersity of functional nanoparticles, however, necessitate additional size assessment to comply with best practices recommendations^10^ and to avoid erroneous interpretation of the DLS data. While electron microscopy methods are increasingly used for such purpose, they often fail to establish correlation with DLS^11–14^, and in many cases they only offer visual confirmation of the nanoparticle assembly^1^ as opposed to quantitative size information. In our view, statistical and reliable distributional data must be part of any size analysis for credible characterization of polydisperse nanoparticle samples using microscopy.

Transmission electron microscopy (TEM) and energy-dispersive X-ray spectrometry (EDX) have proven to be valuable in validation of core-shell structures for a broad range of material systems^15–19^, but their use in the case of polyethylene glycol (PEG) is less applicable. PEG is not visible either in stained TEM images due to its low affinity with uranyl acetate^4^ or in cryo-TEM images due to its low electron density^5^. So, to study and validate the core shell-structure of pegylated polyplexes, we present an alternate measurement approach of combining AFM with DLS and HPLC-MS measurements.

The choice of AFM as a microscopy technique in preference to electron microscopy is twofold in this and in our previous studies^20,21^. It allows observation of biological nanoparticles under fluid conditions and yields statistical size measurements that are quantitative and representative of the undistorted size of the particle population in solution. Microscopy studies often report width measurements without much consideration to the geometry and/or deformation of the particles, and fail to validate that the measurand represents the particle size in solution. This is a major challenge critically affecting the accuracy and reproducibility of imaging data. In this work we address the problem by fixing the otherwise soft polyplex nanoparticles with glutaraldehyde prior to the AFM measurements so that they have a close-to spherical shape. This way their height is representative of their undeformed size. We demonstrate that application of AFM to polyplex imaging and characterization is fully explored and validated, and in combination with DLS and chemical analysis is fully capable of assessing the size and core-shell structure of polyplexes. We obtain a comprehensive spatial description of the nanoparticles to interpret the evolution of size and polydispersity (*i*.*e*., the width of the size distribution) throughout the formulation, and identify regions of the parameter space where stable particle formation occurs. Key aspects of our measurement approach are:

- Method validation through the development of a model system.
- Innovative sample preparation of soft polyplex nanoparticles for statistical and quantitative AFM size analysis.
- Systematic mapping of self-assembly over an extended range of parameter space.
- Investigation of technique-specific response of size measurement methods to polydispersity.
- Adaptability to a variety of charge-driven assemblies of proteins, macromolecules, nucleic acids and nanoparticles.

## Methods

### Gold nanoparticles

A series of negatively charged, citrate-stabilized gold nanoparticles with nominal sizes of 5 nm, 10 nm, 30 nm, 40 nm, 50 nm, 60 nm, 80 nm, 100 nm, 150 nm, 200 nm, and 250 nm served as the core of the model system, and was obtained from Ted Pella Inc., Redding CA^22^. They are chosen for being incompressible and nearly spherical in shape. Gold nanoparticles exhibit a narrow size distribution with relative standard deviation that is constant for the entire 11-member series. They are also chosen for being readily available commercially due to their cost-effective manufacturing. Because of the modular design of the self-assembly platform, gold nanoparticles may be functionalized with the same pegylated polyethylenimine (PEI) system that is used for nucleic acid condensation.

### Polyplex nanoparticles

Two pegylated forms of branched PEI (Mw:25 kDa, Aldrich, USA) were prepared. One with 5-kDa polyethylene glycol (PEG) plus an arginylglycylaspartic acid (RGD) peptide at its distal end, *i*.*e*., RGD-PEG-PEI (RPP), and the other with PEG but without the peptide, *i*.*e*., PEG-PEI (PP). Synthesis of PP and RPP was carried out as reported in Schiffelers *et al*.^23^. Conjugates were dialyzed using 100 kDa molecular weight cutoff dialysis tubing. The average degree of conjugation of PEG to PEI, defined as the percentage of PEI amines conjugated with PEG and PEG-RGD in PP and RPP respectively was determined by proton NMR analysis of the conjugates using the ratio of area under the -CH_2_- proton peaks of PEI (2.8–3.1 ppm) and PEG (3.3–3.6 ppm) (500 MHz spectrometer, Varian). The degree of conjugation was 10% and 7% respectively for the PP and RPP used in this study. Unconjugated PEI was blended with PP or RPP at different molar ratios. Polyplexes were prepared by mixing the polymer blend and nucleic acid—plasmid DNA, 21 bp oligo DNA, or 25 bp siRNA—at different molar ratios of nitrogen (PEI) and phosphate (nucleic acid), *i*.*e*., N/P ratios, over the range of 1 to 4. When required, polyplex nanoparticles were fixed by mixing equal volumes of sample solutions and 0.5% to 1% glutaraldehyde (GA) in phosphate-buffered saline (1x PBS).

### Atomic force microscopy

Well-dispersed individual gold or polyplex nanoparticles are attached by optimizing the surface zeta potential of the substrate and the concentration of the nanoparticle solutions. In particular, a 20-µl droplet of the nanoparticle solution was deposited onto freshly cleaved mica or poly-L-lysine coated mica (PLL-mica) and incubated for 1 minute to 30 minutes. Samples were immersed in water to remove unattached particles and imaged under either fluid or ambient conditions. To obtain a statistically meaningful estimate of size by AFM, several hundred individual nanoparticles are measured for each size. Size information of particles is typically obtained from AFM height data. Before attaching them to the surface, we utilize glutaraldehyde (GA) fixing to preserve the solution shape of polyplex cores, and to enhance their resistance against attachment-induced deformation, drying, and AFM imaging. Based on correlated AFM height and width measurements of subsets of polyplex cores, a shape correction factor of 1.2 and 1.7 was determined for oligo DNA-based and siRNA-based polyplexes, respectively, and was applied to AFM height data of several hundred polyplex nanoparticles for statistical determination of an equivalent-sphere core diameter.

AFM imaging was performed with a Veeco MultiMode AFM and Nanoscope IV controller. Nanoscope Version 6 software was used for data acquisition. Dry imaging was performed in TappingMode using Veeco OTESP cantilevers. For fluid imaging, a TappingMode fluid cell, without an O-ring, and Veeco OTR8 ‘B’ cantilevers (24-kHz nominal resonance frequency in air) were used by oscillating the cantilever in the low-frequency acoustic mode region, ca. 7 kHz to 9 kHz. Particle size analysis using AFM image data was performed using resources available in Nanoscope Version 5 and ImageJ. A first-order image flattening routine is applied to obtain a global background suitable for obtaining particle height measurements. Particles in each image are identified by visual inspection and excluded before the background is calculated. No filtering of the data was performed. The expanded uncertainty calculated at the 95% confidence interval for the AFM height measurements is 2.8%. In addition to repeatability of replicate measurements of the modal height values, other major uncertainty components arise from particle-substrate and particle-tip deformations, background flatness, and calibration. The combined standard uncertainty is estimated then by the square root of the sum of the squares of all the individual uncertainty components.

### Dynamic light scattering

DLS measurements were performed using a ZetaSizer Nano-ZS (Malvern Instruments). Each sample was loaded undiluted into a disposable micro cuvette and measured at 25 °C. Intensity-weighted size distributions and z-average sizes were obtained using the “general purpose” non-negatively constrained least-squares method and the cumulant analysis, respectively^24^. The expanded uncertainty calculated at the 95% confidence interval for the DLS z-average measurements is 1.6%. In addition to repeatability of replicate measurements, other sources of measurement uncertainty for DLS include decay rate, noise and particle interaction. The combined standard uncertainty is estimated then by the square root of the sum of the squares of all the individual uncertainty components.

### High performance liquid chromatography and mass spectroscopy

HPLC-UV and MS measurements were performed with an UltiMate 3000 RSLCnano (Dionex) and micrOTOF II (Bruker), respectively, for quantification of PEG uptake by gold and polyplex nanoparticles, and for measurements of the nucleic acid uptake by polyplexes. A mixture of acetonitrile and distilled water (80:20, v/v) containing 0.1% formic acid was used as the mobile phase. The flow rates were adjusted to 5 μL/min and 300 nL/min for loading and measurement, respectively. Acclaim PepMap100 C18 (3 μm) 75 μm x 15 cm analytical, and Acclaim PepMap100 C18 (5 μm) 100 μm x 1 cm trap columns were used. Nanoparticles are separated from the supernatant by centrifugation. PEG uptake is only measured for nanoparticles complexed with RPP through the detection of its UV-sensitive fragment (GDMFGCA). This RGD chromophore is released from the RPP macromolecule by sequential trypsin and dithiothreitol (DTT) or tris(2-carboxyethyl) phosphine (TCEP) digestion and identified by mass spectroscopy at a MW of about 700. Elution conditions for pre-concentration setup and UV detection at 190 nm are then optimized for the chromophore, and a UV calibration curve is obtained for the RGD concentration. The amount of nucleic acid incorporated into polyplexes is determined by HPLC-UV direct injection measurements at 260 nm. Polyplexes are separated first and the supernatant is checked for free DNA utilizing ESI-MS in negative ion mode. If DNA is present, the spectrogram exhibits numerous multiply charged species generated by different amount of deprotination and adduct formation. Polyplexes are then dissociated in heparin (0.2 mg/mL) and high pH (100 mM NaOH). The amount of DNA is quantified using a standard calibration curve.

## Results and Discussion

**Figure 1:**
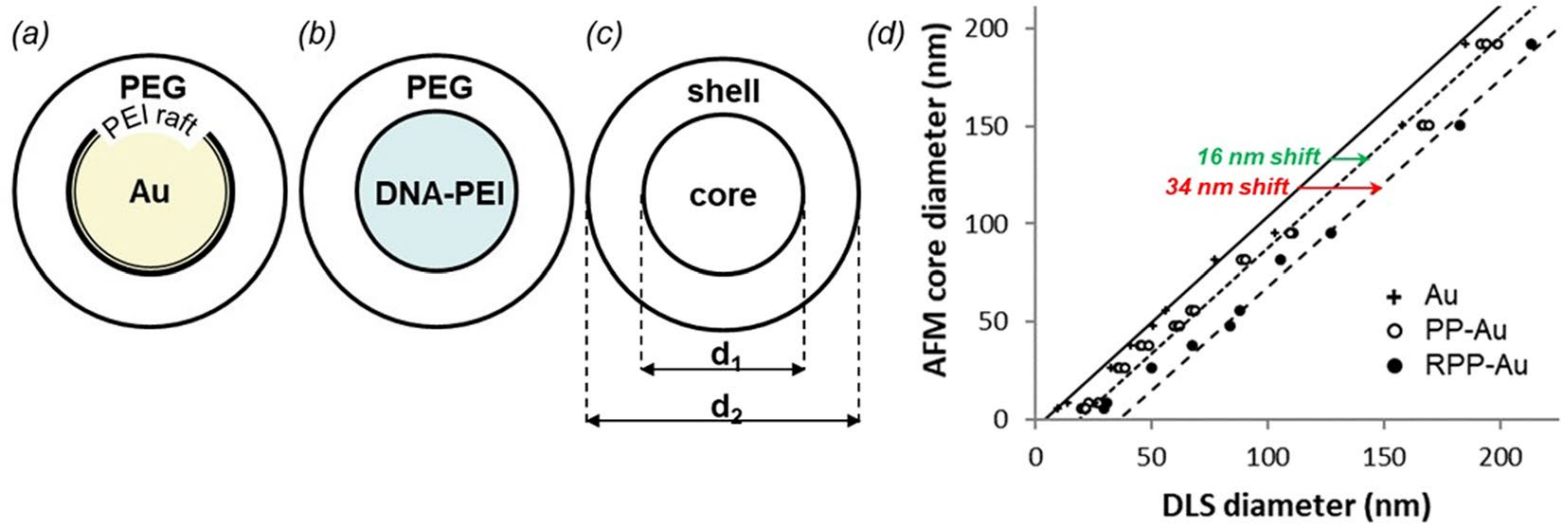
Schematic representation of (**a**) the gold nanoparticle model system, (**b**) the self-assembled polyplexes of nucleic acid and polycationic macromolecules and (**c**) the core-shell structure. An example of a measurement set for a single particle size. Shell thickness equals ½ (d_2_-d_1_). (**d**) Correlated core diameter and hydrodynamic diameter of functionalized gold nanoparticles measured by AFM and DLS, respectively. Data points represent statistically averaged measurements.

The formulation platform employed in this work has been developed with a modular approach to structure and functionality^23^. Nanoparticles are self-assembled into a well-defined payload-carrying core and a steric protective shell as shown in Fig. 1b, optionally decorated with exposed targeting molecules (not shown). This nanoparticle system was shown to be functionally active *in vivo*, demonstrated by the RGD ligand mediated delivery of anti-angiogenic siRNA to tumor and ocular angiogenic animal models leading to inhibition of angiogenesis^23,25^. Self-assembly of the components is charge-driven, hence the platform’s adaptability to a variety of macromolecules and nanoparticles. The modular design allows substitution of constituents with similar physical-chemical properties, as well as optimization for functional requirements. For negatively charged payloads, like nucleic acids, polycationic macromolecules are effective complexing agents. The electrostatic interactions between these components results in polyplex formation. Because the resulting nanoparticles are positive, a steric PEG shell is utilized to minimize non-specific interactions with cells, to prevent protein binding, and to improve stability.

Dimensional metrology of such core-shell particles must include size measurements of the core as well as the shell. We propose to measure the core by AFM, and to measure the hydrodynamic size of the particle—*i*.*e*., the core and the shell together—by DLS, per Fig. 1c. These measurements are particularly complicated for polyplexes as their core and shell are made up of very similar macromolecules. Systematic mapping of the self-assembly over an extended range of parameter space is required first in order to confirm the appropriateness of the ideal core-shell structure proposed in Fig. 1b. Additional measurement challenges are introduced by the polydispersity of the polyplexes. DLS and AFM measurements have biases toward different parts of a broad size distribution; even small tailing of the distribution to larger sizes is sufficient to shift the DLS diameter significantly. Therefore, method development and validation through a model system—with a nearly ideal size distribution and core-shell structure—is a prerequisite.

### Model system

The schematic of the model system—consisting of a series of gold nanoparticles between sizes of 5 nm to 250 nm—is shown in Fig. 1a. The polymer system is prepared by covalent conjugation of 5-kDa PEG molecules to multiple sites on a branched 25-kDa PEI macromolecule raft. This approach to functionalization utilizes electrostatic wrapping of polyelectrolytes onto the oppositely charged gold surfaces^26,27^ without the specificity of chemical coupling reactions such as thiolation. The structural and molecular arrangement of PEG chains determines the thickness and grafting density of the shell, and consequently the stability of the nanoparticles.

Shell thickness of the model system is obtained from combining DLS and AFM measurements per Fig. 1c for each member of the gold nanoparticle series. First, the size of the unfunctionalized gold nanoparticles—*i*.*e*., the core—is measured by DLS and AFM. For the latter, individual gold nanoparticles are attached onto PLL-mica substrates and typically several hundred individual gold nanoparticles are measured for each size. Next, the gold nanoparticle series is functionalized with PP or RPP under a highly-saturated condition and re-measured by DLS. For each gold nanoparticle size, AFM measurements are correlated then with the three DLS measurements pairwise. The resulting three datasets are shown in Fig. 1d. For the series of unfunctionalized gold nanoparticles there is a roughly 1 to 1 relationship between AFM and DLS sizes. This relation serves as a reference, against which the functionalized gold nanoparticles are compared.

The hydrodynamic size increases for both the PP and RPP-functionalized gold series, which is independent of core sizes until curvature effects become significant at small diameter^28^. With increasing curvature, the volume available for the adsorbed PEG macromolecules increases. For the same volume, the thickness becomes smaller with increasing curvature with respect to the flat surface. The larger the PEG density, the bigger the curvature effect is. According to Eq. 6 of Ref.^28^, the curvature effect is expected to become noticeable in our case for nanoparticles smaller than 50 nm. The results in Fig. 1d agree. The shape and width of the intensity-weighted DLS diameter distributions remain unchanged after functionalization. We propose that the size increase is due to the PEG shell, the thickness of which is determined then as half the shift between DLS and AFM sizes. Because of uncertainties in particle shape, and size, and possible interferences caused by plasmon resonances, the shell thickness was determined from systematic shifts between trendlines, as shown in Fig. 1d, based on the entire particle series. Accordingly, the thickness of the PP and RPP shells are estimated at 8 nm and 17 nm, respectively. Although the difference in shell thickness may be simply due to the size difference between PEG and PEG-RGD conjugated to PEI, we suggest an alternative explanation. Chemical modification with the RGD peptide may also alter the coordination and steric shielding capability of the macromolecules, as well as their interaction with each other and the negatively charged surface of gold nanoparticle cores. As a result, the PEG-RGD chains may conform to a denser brush configuration. To investigate this point, the relationship between shell thickness and grafting density must be determined next.

**Figure 2:**
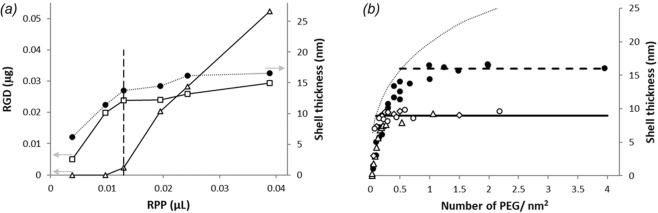
(**a**) Shell thickness (solid circles) and partitioning of PEG between uptake (open squares) and excess in supernatant (open triangles) as a function of RPP solution volume mixed with 100 nm gold nanoparticles. The concentration of the RPP and gold solutions is 8.3 µg/µL and 5.6 × 10^6^ particles/uL, respectively. (**b**) Shell thickness as a function of PEG grafting density for 30 nm (triangles), 80 nm (diamonds) and 100 nm (circles) gold nanoparticles functionalized with PP (open symbols) and RPP (solid symbols) systems. Horizontal lines correspond to the equilibrium shell thicknesses. The dotted line shows the theoretical prediction^29^ between PEG density (D) and shell thickness (L), calculated as $L=N{a}^{5/3}{D}^{1/3}$, where *N* = 114 and *a* = 0.35 *nm* for the 5-kDa PEG.

PP or RPP solution is sequentially added to gold nanoparticles of selected sizes and their hydrodynamic size and PEG uptake are measured by DLS and HPLC-UV, respectively. The results, shown in Fig. 2a for 100 nm gold nanoparticles, reveal that the increase in shell thickness parallels PEG uptake by the nanoparticles and both plateau concurrently. The PEG uptake is nearly complete until the shell thickness reaches its maximum. Further addition of PEG ends up in the supernatant. Assuming complete uptake at saturation point, the number of PEG per unit area is estimated from i) the amount of PEG mixed with the gold nanoparticles ii) the AFM core size and iii) the concentration of the gold nanoparticle solutions as provided by the manufacturer. The results are shown in Fig. 2b along with theoretical prediction for shell thickness as a function of PEG grafting density^29^. While the assumption of complete uptake is reasonable until the shell thickness reaches its maximum, it provides an upper estimate for the experimentally calculated coverage, slightly overestimating the PEG grafting density with respect to the theoretical expectation. At the end of the sequential addition of PP and RPP, equilibrium shell thicknesses are like those obtained for the all-at-once addition of the corresponding solutions in Fig. 1d.

The shell thickness of the model system functionalized with PP plateaus at a grafting density of about 0.15 PEG/nm^2^, which is comparable to what has been reported for thiolated grafting of the same size PEG onto gold nanoparticles^30^. In comparison, modification of PP with the RGD peptide allows adsorption of more PEGylated macromolecules onto the gold nanoparticles yielding a higher coverage of about 0.5 PEG/nm^2^. Comparable adsorption efficiency was reported by the method of ‘living’ PEGylation, where gold nanoparticles are synthetized without a citrate capping layer to enhance the uptake of thiolated PEG molecules^31^.

The results of the investigation into the core-shell structure of the model system and molecular arrangement of its PEG shell provide the necessary framework to now transition the measurement methods to more complex core-shell assemblies of macromolecules.

### Core-shell polyplex nanoparticles

There are several key factors—both chemical and structural—to the construction of functional polyplexes. Core formation depends on the types of nucleic acid and polycationic macromolecules, their charge ratio and relative size. In our experience, the size of the complexing macromolecule is especially important for small (less than 25-mer) nucleic acids as they could only be complexed with sufficiently large polycations. Conjugation of PEG to polycationic macromolecules prior to complexation is preferred over PEGylation of the pre-formed cores for two reasons. First and foremost, PEG plays an active role in the self-assembly process as demonstrated in the forthcoming results. Second, initial attachment of PEG chains to the pre-formed cores sterically hinders subsequent addition and limits the grafting density.

Several studies have concluded that long, at least 5-kDa, PEG chains are preferred for optimal shielding^32,33^. High grafting density is also critical to avoid aggregation of the polyplexes, and to prevent their interaction with blood components and cells. Such conjugates however exhibit poor condensation properties. Interference from many PEG chains results in non-spherical, loose polyplex structures and instead forces the use of macromolecules with a low number of attached PEG. One way to achieve sufficient condensation as well as a dense protective shell in a single self-assembly step is the substitution of some of the PEGylated polycations with unconjugated counterparts^23,34^. This approach has also been investigated for mixed PEI and PEG-PLL systems^35^ with the demonstration of combined benefits of PEG stabilization and PEI condensation capabilities. In the present work, PEG is covalently and randomly conjugated to multiple sites on branched PEI, and then mixed with unconjugated PEI at different molar ratios (PEI/PP) in terms of amine concentration.

The next sections explore the formulation space of the self-assembly with respect to stability and investigate the effect of three parameters—N/P ratio, PEI/PP ratio and degree of PEG conjugation—on core-shell formation and the size and functionality of polyplex nanoparticles.

### N/P ratio

Branched PEI bears positive charges on the primary, secondary and tertiary amines facilitating strong electrostatic interaction with the negatively charged phosphate groups of nucleic acids^36^. Since this is the interaction that drives the self-assembly process, N/P ratio—defined as the molar ratio of PEI nitrogen and DNA phosphate—is a predominant formulation parameter^37,38^.

**Figure 3:**
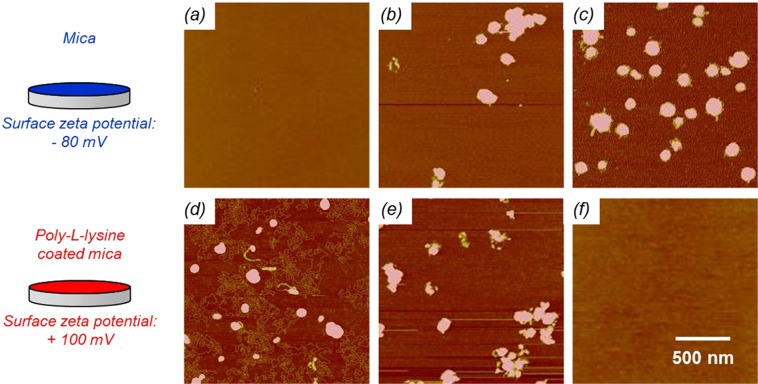
AFM topographic images (5 nm Z-scale) of plasmid DNA-based polyplex nanoparticles formed at N/P ratios of 2, 3, and 4 adsorbed onto mica (**a**–**c**) and PLL-mica (**d**–**f**), respectively.

Plasmid DNA condensation and surface charge of the resulting polyplexes are studied as a function of N/P ratio with AFM. Negative mica and positive PLL-mica substrates are used to immobilize polyplexes for AFM imaging under ambient condition. At low N/P ratios, polyplexes are negatively charged, therefore they exclusively adsorb onto the positive surface (Fig. 3d). Naked plasmid DNA are visible, indicating partial condensation. Around electroneutrality, polyplexes tend to aggregate and adsorb onto both negative and positive surfaces (Fig. 3b,e). DNA condensation is complete at this point as no free plasmid DNA is observed. At high N/P ratios, polyplexes are positively charged, therefore they exclusively adsorb onto the negative surface (Fig. 3c). We conclude that electroneutrality—where PEI completely binds the DNA and the zeta potential of the particles shifts from negative to positive—occurs between N/P ratios of 2 and 3 for the plasmid DNA-based polyplex.

For oligo DNA and siRNA-based polyplexes, complete condensation occurs at an N/P ratio of 2 determined by mass spectroscopy. The lower value is attributed to the fact that more PEI macromolecules are required to neutralize plasmid DNA than siRNA or oligo DNA because of three-dimensional conformational constraints. According to molecular dynamics simulations on the complexation between linear PEI and DNA or siRNA^39^, more protonated PEI nitrogen is required to cover the phosphate groups of plasmid DNA than siRNA. This is because the phosphate groups of siRNA are closer together than those in plasmid, and therefore the likelihood of a PEI nitrogen to interact with two different phosphate groups at the same time is significantly higher for siRNA^39^. Tendency for aggregation is high at N/P ratios close to electroneutrality. For increased stability and optimal condensation, we choose to keep the N/P ratio constant at 4 for the investigation of the PEI/PP parameter space next.

### PEI/PP ratio

DLS is probably the most common method to monitor polyplex size during formulation. AFM is less common but essential—as we demonstrate here—for confirming particle formation and mapping out the parameter space for optimal self-assembly.

**Figure 4:**
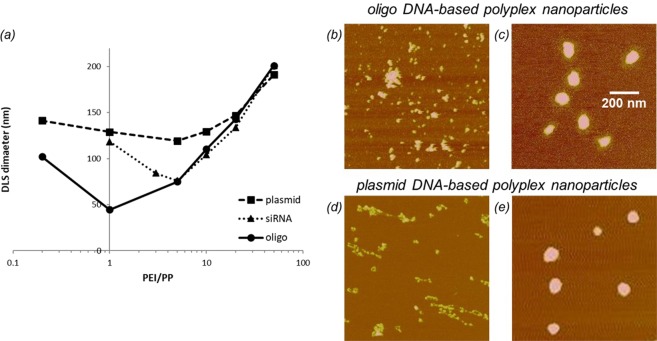
(**a**) DLS size measurements of nucleic-acid-based formulations as a function of PEI/PP ratio. (**b**–**e**) AFM topographic images (20 nm Z-scale) of oligo-, and plasmid DNA-based polyplex nanoparticles formed at PEI/PP ratios of 1:5 (**b**,**d**), and 10:1 (**c**,**e**). Note the extended morphology and lack of condensation of the plasmid DNA complex in (**d**).

Hydrodynamic size measured by DLS is shown in Fig. 4a for different nucleic-acid-based polyplexes as a function of PEI/PP ratio. Polyplex size exhibits a minimum and increases both to the low and high end of the PEI/PP parameter space. Based on the DLS results only, there is no discernable size difference between polyplexes formed at 1:5 and 10:1 PEI/PP ratios in either case of the oligo or plasmid DNA-based systems. Cross-examination by AFM, however, shows that particle formation is very different at these PEI/PP ratios. At 10:1, stable particles are formed for both the oligo and plasmid DNA systems as shown respectively in Fig. 4c,e. In contrast, association of components with a large degree of aggregation or networking occurs at PEI/PP ratio of 1:5. Representative examples of poorly formed, unstable polyplexes are displayed in Fig. 4b,d for the oligo and plasmid DNA systems, respectively.

With complementary AFM assessment of the particle formation, further interpretation of the DLS results in Fig. 4a is possible. The minimum particle size, which corresponds to encapsulation of the smallest number of DNA, is larger for the plasmid than for the oligo DNA-based systems. This means that plasmid DNA interacts with more PEI macromolecules for complete condensation than oligo DNA does. At high PEI/PP ratios, size tends to be the same for all polyplexes. Only at low PEI/PP ratios is the self-assembly affected by differences in the nucleic acid structure. The parameter space for stable particle formation is narrower for siRNA than for either the plasmid or oligo DNA-based systems. These results identify the optimal PEI/PP region for further exploration of the core-shell structure and for investigation of the differences in complex formation between the oligo DNA and siRNA systems.

### Nucleic acid-PEI core

The design of the model system allows measurements of its core before as well as after functionalization with PEG. Examination of the nucleic acid-PEI cores of the polyplexes is not possible before PEGylation since they only exist in the form of a core-shell assembly. It is coincidentally possible however to measure the core of fully formed polyplexes with AFM. While imaging functionalized gold nanoparticles, we find that the brush-like PEG chains have little resistance against the imaging force typically applied by an oscillating AFM tip. All information contained in the topographic AFM image is therefore of the much denser core of the polyplexes. Depending on their bending rigidity and interaction with substrates, polyplex cores may deform to different degrees upon attachment for AFM imaging^20^. Consequently, sample preparation and imaging conditions are key for AFM measurements^21^.

**Figure 5:**
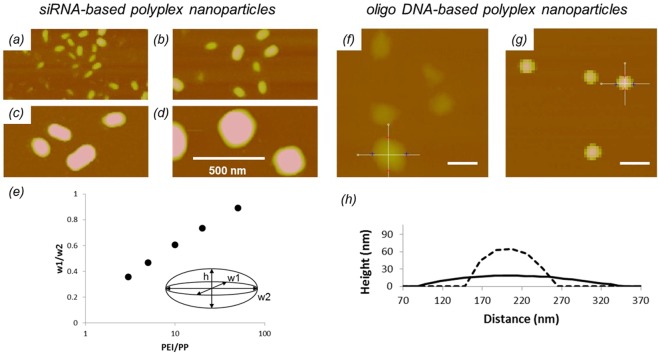
(**a**–**e**) SiRNA-based polyplexes nanoparticles. AFM topographic images (50 nm Z-scale) of siRNA-based polyplex cores formed at PEI/PP ratios of (**a**) 5:1, (**b**) 10:1, (**c**) 20:1, and (**d**) 50:1. (**e**) Ratios of perpendicular width measurements of siRNA-based ployplex cores as a function of PEI/PP ratio. (**f**–**h**) Oligo DNA-based polyplex nanoparticles. AFM topographic images (150 nm Z-scale) of oligo DNA-based polyplex cores that were formed at a 20:1 PEI/PP ratio and adsorbed (**f**) unfixed and (**g**) fixed onto mica substrates. Scale bar: 200 nm. (**h**) Representative cross sections of unfixed (solid line) and fixed (dashed line) oligo DNA-based polyplex cores.

The size of the oligo DNA and siRNA employed in this work is about the same, but the differences in their conformational adaptability allow them to interact with macromolecules very differently^40^. To study the morphology and fluidity of the cores they form, polyplexes are immobilized on mica and imaged by AFM under fluid condition (Fig. 5). Shape information is obtained by measuring the core size in three perpendicular directions according to the inset in Fig. 5e. Ratios of the width measurements for the siRNA-based polyplex cores are plotted in Fig. 5e. The cores exhibit an ellipsoidal shape, which evolves toward spherical configurations with increasing PEI/PP ratio. They are solid-like and their shape remains unchanged even after fixing, details of which are provided later in this section. In comparison, oligo DNA-based polyplex cores are fluid-like. Figure 5f,h indicate that they are highly distorted into circular patches once adsorbed onto mica. When fixed in solution prior to attachment, they appear to be nearly spherical (Fig. 5g,h). These results are consistent with the fact that RNA has a larger persistence length than DNA does^41,42^, and is expected to form rigid rods. It is the rigidity that accounts for the observed differences in complex formation between the siRNA and oligo DNA systems seen in Fig. 4a.

Size information of particles is typically obtained from AFM height data. Accordingly, measurements of soft nanoparticles require innovative sample preparation and considerations of deposition-induced deformation in AFM image analysis^21,43^. In this work, we utilize the high amine density of PEI for cross-linking with glutaraldehyde (GA)^44,45^. GA fixing is expected to preserve the solution shape of the polyplex cores, and to enhance their resistance against attachment-induced deformation and drying. As seen in Fig. 5, GA-fixed oligo DNA-based cores are nearly spherical. DLS shows that no structural change—*i*.*e*., shrinkage—occurs to the polyplexes due to fixing. The fact that cores are not perturbed by cross-linking implies that the complexation between DNA and PEI is strong to begin with. AFM measurements show no size difference between unfixed and fixed siRNA-based polyplexes, so GA does not increase the resistance of the shell against the imaging force. With that, AFM height measurements of GA-fixed polyplexes are now validated to provide reasonable estimates for their core size in solution. The evolution of core sizes over an extended range of the parameter space is investigated next.

**Figure 6:**
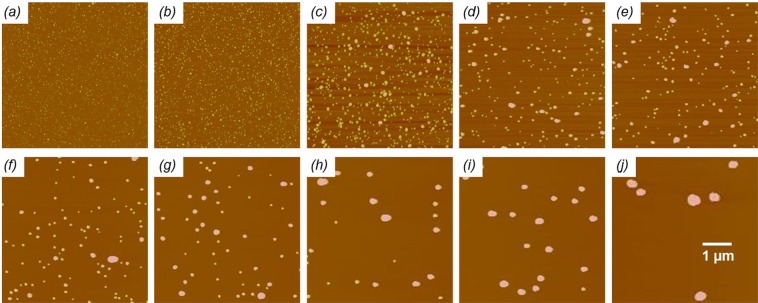
AFM topographic images (100 nm Z-scale) of oligo DNA-based polyplex nanoparticles formed at PEI/RPP ratios of 1:1 (**a**), 3:1 (**b**), 5:1 (**c**), 8:1 (**d**), 10:1 (**e**), 12:1 (**f**), 15:1 (**g**), 20:1 (**h**), 30:1 (**i**), and 50:1 (**j**). Polyplex nanoparticles are fixed prior to attachment to mica substrates and imaged under ambient condition. The brush-like PEG chains have little resistance against the imaging force typically applied by an oscillating AFM tip. All information contained in the topographic AFM image is therefore of the much denser core of the polyplex nanoparticles.

Series of polyplexes are prepared by complexing nucleic acids with both PP and RPP at dozen of different PEI/(R)PP ratios while keeping the N/P ratio constant at 4. Representative AFM topographic images of the RPP-oligo DNA series are shown in Fig. 6. No aggregation is seen for the investigated PEI/RPP parameter space. Stable polyplexes with core sizes as small as 10 nm are observed at the PEI/RPP ratio of 1 and N/P ratio of 4. This is significant because without the use of PEGylated components a very high N/P ratio of 20 is required to achieve stable formation of similarly small particles^46^. The size of the cores as well as the broadness of their distribution, *i*.*e*., polydispersity, are progressively increasing with PEI/RPP ratio. When core sizes exceed 100 nm, the polydispersity seems to decrease again.

### Shell thickness

**Figure 7:**
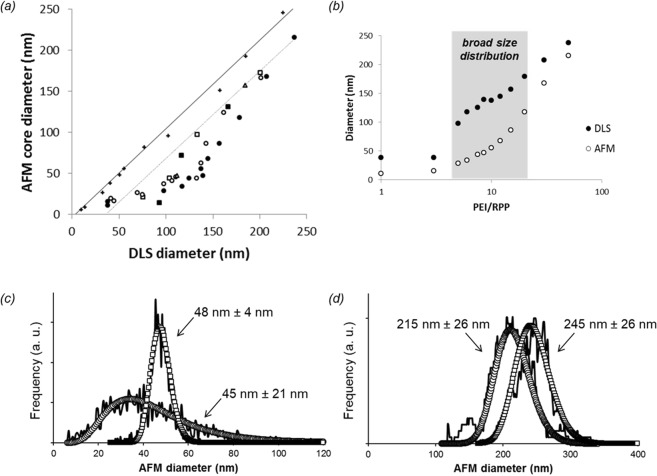
(**a**) Correlated core diameter and hydrodynamic diameter of core-shell nanoparticles measured by AFM and DLS, respectively. Gold (+), PP-oligo (○), RPP-oligo (●), PP-siRNA (□), RPP-siRNA (■), and PP-plasmid (∆). (**b**) AFM core and DLS hydrodynamic diameters of oligo-based polyplex nanoparticles as a function of PEI/RPP ratio. (**c**) Comparison of AFM core size distributions of 50 nm gold nanoparticles and oligo-based polyplex nanoparticles formulated at a 10:1 PEI/PP ratio. (**d**) Comparison of AFM core size distributions of 250 nm gold nanoparticles and oligo-based polyplex nanoparticles formulated at a 50:1 PEI/RPP ratio. Open circles/squares represent log-normal fits to the AFM distributions obtained from imaging data of individual nanoparticles.

Shell thickness of the polyplexes is measured by combining DLS and AFM per Fig. 1c. The results for five polyplex series—prepared at an N/P ratio of 4 over a broad size range by varying PEI/(R)PP ratios—are compared with the unfunctionalized gold reference in Fig. 7a. Unlike for the model system, the shift between DLS and AFM sizes is not constant across the polyplex series. When the data is re-plotted as a function of the PEI/(R)PP ratio in Fig. 7b, it becomes apparent that the size difference between DLS and AFM varies in the middle of the parameter space where polydispersity of the cores is also high. For example, core size distribution at 10:1 PEI/(R)PP ratio is significantly broader than that of similarly sized gold nanoparticles (Fig. 7c). At 50:1 on the other hand, the distribution of core sizes is comparable to that of the model system (Fig. 7d). Deviation from the straight line in Fig. 7a is due to the different response of the DLS and AFM methods to polydispersity. Therefore, only data points corresponding to polyplexes with relatively narrow size distribution are selected for estimation of the shift between DLS and AFM sizes. From such shift, indicated by the dotted line in Fig. 7a, a constant shell thickness of 17 nm is determined for all polyplexes. This estimate is comparable to what is obtained for the RPP-functionalized model system. Note that for polyplexes smaller than 50 nm the curvature effect becomes noticeable as discussed earlier for the gold nanoparticles.

**Figure 8:**
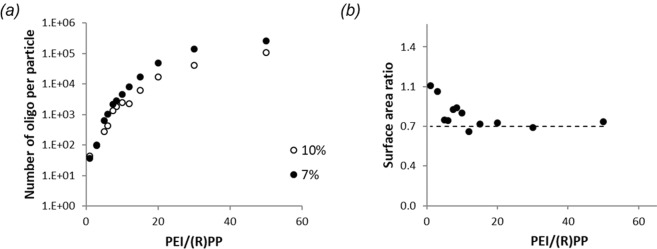
(**a**) Number of oligo encapsulated within the core of polyplex nanoparticles as a function of PEI/(R)PP ratio at 7% and 10% degrees of conjugation. (**b**) Total surface area ratio of the system of polyplex nanoparticles formed at 7% and 10% degrees of conjugation as a function of PEI/(R)PP ratio. The horizontal line represents a total surface area ratio of 0.7.

So far, we have systematically investigated the formation of core-shell polyplex nanoparticles and gathered dimensional information about them by DLS and AFM. Next, these results are combined with molecular analysis. The amount of nucleic acid incorporated into the cores is determined by HPLC-UV as described in the Methods section. To determine the number of DNA per particle, calculations assume the AFM core sizes from Fig. 7a, a MW of 12850 for the oligo, and a core density of 1370 µg/µL as an approximation based on protein compressibility measurements^47^. The results for the oligo DNA system are shown in Fig. 8a as a function of PEI/(R)PP ratio for two different degrees of (R)PP conjugation. The amount of DNA per particle is set not only by the ratio of the unconjugated and PEGylated PEI, but by the number of PEG chains per PEI as well.

### Grafting density

The mechanism of shell formation is fundamentally different for the polyplex and model systems. For the latter, grafting of PEGylated macromolecules onto gold nanoparticles is limited by steric hindrance from the already adsorbed PEG chains. Minimizing the steric footprint of the PEG chains—with an RGD peptide as done in this work or by partially collapsing them by solvent conditions^48^—significantly increases the PEG grafting density on nanoparticles. In case of the polyplex self-assembly, formation of the shell is driven by interactions of the PEG-conjugated PEI with nucleic acid and is not dependent on penetration of unattached PEG through a progressively forming shell. According to the results in Fig. 7a, shell thickness of the polyplexes is independent of whether or not the PEG has an RGD peptide at its distal end. This suggests that the condensation—between the oppositely charged PEI and nucleic acid components—continues until an equilibrium PEG grafting density is reached on the surface of the polyplex cores.

In the case of such a limiting mechanism, a direct relationship is expected between the number of PEG chains conjugated to PEI macromolecules and the total surface area of the polyplex cores. To show that this is indeed the case, we calculate the ratio of the total surface area of polyplex cores formed at 7% and 10% degrees of conjugation. The N/P ratio is fixed at 4. Keeping the amount of oligo used for complexation—hence the total core volume—the same, the total surface area ratio is equal to the inverse of the ratio of core diameters measured by AFM for the aforesaid polyplexes. The result is plotted in Fig. 8b as a function of PEI/(R)PP. The total surface area ratio tends to 0.7, which is equal to the conjugation ratio of the two systems. This means that condensation by PEI rafts with lower number of PEG chains produces larger cores for a decreased total surface area so that the equilibrium PEG grafting density is converging to the same value for all polyplexes. Deviations from 0.7 at low PEI/(R)PP most probably are due to high levels and/or different amounts of excess PEI and PEGylated PEI remaining in solution^49^.

As demonstrated for the model system, estimation of the equilibrium PEG grafting density is possible from AFM core size and HPLC_UV measurements. The polyplex solution prepared at 50:1 PEI/(R)PP ratio is an ideal candidate for such investigation for several reasons. First, the large particle size ensures clean separation of the supernatant for HPLC-UV. Second, narrow size distribution of the cores ensures reliable determination of the shell thickness from correlated DLS and AFM measurements. HPLC-UV and MS measurements reveal no free RPP or oligo in the supernatant, confirming full incorporation of both the PEGylated and nucleic acid components into the polyplexes. From the amount of PEG used during complexation and the total surface area of the cores—which is calculated with the assumptions of complete oligo uptake, a 1370 µg/µL core density^47^, and the AFM core size—the equilibrium PEG grafting density is estimated to be 0.5 PEG/nm^2^. Based on theoretical predictions and data from the RPP-functionalized model system, the 0.5 PEG/nm^2^ grafting density agrees with the 17 nm shell thickness determined for all polyplexes in Fig. 7a. We argue that reaching equilibrium at this grafting density limits the core formation of all polyplexes investigated in this work.

## Conclusion

A combined dimensional and analytical chemical measurement approach has been developed and validated to characterize self-assembled polyplexes with core-shell structure. The use of a model system is critically important in this work for interpretation of the multi-technique measurement data, especially for distinguishing the notion of size dependence from non-ideal distributional properties, such as polydispersity. By providing quantitative size, structural and compositional information, the results presented here have led to an improved understanding of the self-assembly process and of the active role the PEGylated component plays in the core-shell formation. Stable core-shell polyplex formation has been achieved over a broad size range of about 30 nm to 250 nm by optimizing the N/P and PEI/(R)PP ratios of the self-assembly. The relative polydispersity of these polyplexes inherently varies with size and tends to be smaller as the particles get larger. The polyplex cores possess a varying degree of fluidity depending on the structural property of their nucleic acid payload but have a constant shell thickness of 17 nm in any case.

It has been shown that the PEG grafting density is not controlled by, but itself controls the self-assembly. Neither adjusting the number of PEG conjugated to PEI or the PEI/(R)PP ratios alters the equilibrium grafting density of 0.5 PEG/nm^2^. This value is an intrinsic property of our formulation. Once the equilibrium grafting density is reached, the growth of the polyplex cores terminates. The 0.5 PEG/nm^2^ grafting density is uniquely the same for all nucleic acid-based systems investigated in this work and is significantly higher than obtained for more commonly used PEGylation strategies.

## Data Availability

The datasets generated and analyzed during the current study are available from the corresponding author on reasonable request.
